# Ethoxy­carbonyl­methyl ursolate

**DOI:** 10.1107/S1600536808039706

**Published:** 2008-11-29

**Authors:** Wei Yang, Hua-ling Luo, Cong-ling Yang, Shu-fan Yin, Ying Li

**Affiliations:** a College of Chemistry, Sichuan University, Chengdu 610064, People’s Republic of China

## Abstract

The title compound, C_34_H_54_O_5_, was synthesized by the reaction of ursolic acid with ethyl chloro­acetate in the presence of DMA. All six-membered rings of the penta­cyclic triterpene skeleton adopt chair conformations. In the crystal structure, mol­ecules are linked by inter­molecular O—H⋯O hydrogen-bond inter­actions, forming zigzag chains along the *c* axis.

## Related literature

For the pharmacological activity of ursolic acid, see: Es-saady *et al.* (1996 ▶); Kashiwada *et al.* (2000 ▶). For the crystal structure of ursolic acid, see: Simon *et al.* (1992 ▶). For the synthesis and characterization of the title compound and other ursolic acid derivatives, see: Yang *et al.* (2008 ▶); Liu *et al.* (2007 ▶).
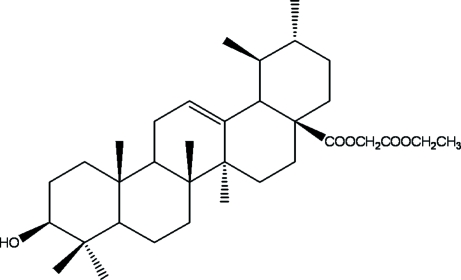


## Experimental

### 

#### Crystal data

C_34_H_54_O_5_
*M* *_r_* = 542.77
Orthorhombic, 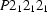

*a* = 11.624 (3) Å
*b* = 12.465 (4) Å
*c* = 21.478 (3) Å
*V* = 3112.0 (14) Å^3^
*Z* = 4
Mo *K*α radiation
μ = 0.07 mm^−1^
*T* = 292 (2) K
0.60 × 0.56 × 0.44 mm

#### Data collection

Enraf–Nonius CAD-4 diffractometer
Absorption correction: none
3435 measured reflections
3116 independent reflections
1818 reflections with *I* > 2σ(*I*)
*R*_int_ = 0.009
3 standard reflections every 200 reflections intensity decay: 0.8%

#### Refinement

*R*[*F* ^2^ > 2σ(*F* ^2^)] = 0.047
*wR*(*F* ^2^) = 0.125
*S* = 0.97
3116 reflections
362 parameters
H-atom parameters constrained
Δρ_max_ = 0.19 e Å^−3^
Δρ_min_ = −0.17 e Å^−3^

### 

Data collection: *DIFRAC* (Gabe & White, 1993 ▶); cell refinement: *DIFRAC*; data reduction: *NRCVAX* (Gabe *et al.*, 1989 ▶); program(s) used to solve structure: *SHELXS97* (Sheldrick, 2008 ▶); program(s) used to refine structure: *SHELXL97* (Sheldrick, 2008 ▶); molecular graphics: *ORTEP-3 for Windows* (Farrugia, 1997 ▶); software used to prepare material for publication: *SHELXL97*.

## Supplementary Material

Crystal structure: contains datablocks global, I. DOI: 10.1107/S1600536808039706/rz2269sup1.cif

Structure factors: contains datablocks I. DOI: 10.1107/S1600536808039706/rz2269Isup2.hkl

Additional supplementary materials:  crystallographic information; 3D view; checkCIF report

## Figures and Tables

**Table 1:** Hydrogen-bond geometry (Å, °)

*D*—H⋯*A*	*D*—H	H⋯*A*	*D*⋯*A*	*D*—H⋯*A*
O1—H1⋯O3^i^	0.82	2.14	2.944 (4)	165

Symmetry code: (i) 
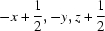
.

**Fig. 1.**
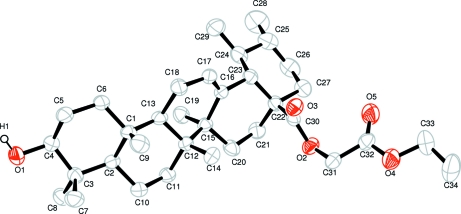
The molecular structure of the title compound, with displacement ellipsoids drawn at the 30% probability level.

## supplementary crystallographic information

### Comment

Ursolic acid, a pentacyclic triterpene existing abundantly in the plant kingdom, has been reported to possess pharmacological activities, such as anti-tumor, anti-inflammatory and anti-HIV activities (Es-saady *et al.*, 1996; Kashiwada *et al.*, 2000). The synthesis and characterization of some derivatives of this compound have been recently reported (Liu *et al.*, 2007). We report herein the crystal structure of the title compound, whose synthetic method has been already reported elsewhere (Yang *et al.*, 2008).

In the molecule of the title compound (Fig.1) bond lengths and angles within the six-membered rings are very similar to those given in the literature for ursolic acid (Simon *et al.*, 1992). The C(sp^2^)—C(sp^2^) bond distance (C16—C17) is 1.317 (5) Å, the C(sp^3^)—C(sp^3^) bond lengths range from 1.506 (6) Å to 1.592 (5) Å, and the three C(sp^3^)—C(sp^2^) bond distances (C17—C18, C16—C23 and C16—C15) are 1.491 (5), 1.528 (5) and 1.545 (5) Å, respectively. The five six-membered rings adopt a chair conformation. The carboxy group at C22 and the methyl groups C7, C9, C14 and C19 are axially oriented, while the hydroxy groups and the methyl groups C8, C28 and C29 are in equatorial positions. The O2–C30–C22–C27 and C2–C30–C22–C21 torsion angles are -72.1 (4) Å and 47.8 Å, respectively. In the crystal packing, intermolecular O—H···O hydrogen bonds (Table 1) link molecules to form zig-zag chains extending along the *c* axis.

### Experimental

To a solution of ursolid acid (456 mg, 1 mmol) in DMF (10 ml) was added K_2_CO_3_ (300 mg, 2.2 mmol), KI (50 mg, 0.3 mmol) and NEt_3_ (0.5 ml). After stirring at room temperature for 10 minutes, a solution of ethyl chlorocetate (0.4 ml, 3 mmol) in DMF was added dropwise, and the reaction monitored by TLC. After completion of the reaction, satured brines and ehtyl acetate were added. The organic layer was separated, washed with water until neutral, dried with Na_2_SO_4_, filtered and evaporated *in vacuo* to get the solid title compound. Colourless crystals suitable for X-ray analysis were obtained by slow evaporation of an ethanol solution at room temperature.

### Refinement

H atoms were positioned geometrically and refined using a riding model, with C—H = 0.93–0.98 Å, O—H = 0.82 Å and with *U*_iso_(H) = 1.2*U*_eq_(C) or 1.5 *U*_eq_(C, O) for hydroxy and methyl H atoms. In the absence of significant anomalous dispersion effects, Friedel pairs were merged.

### Crystal data

**Table:**

C_34_H_54_O_5_	*F*_000_ = 1192
*M**_r_* = 542.77	*D*_x_ = 1.158 Mg m^−^^3^
Orthorhombic, *P*2_1_2_1_2_1_	Mo *K*α radiation λ = 0.71073 Å
Hall symbol: P 2ac 2ab	Cell parameters from 14 reflections
*a* = 11.624 (3) Å	θ = 4.3–5.6º
*b* = 12.465 (4) Å	µ = 0.08 mm^−^^1^
*c* = 21.478 (3) Å	*T* = 292 (2) K
*V* = 3112.0 (14) Å^3^	Block, colourless
*Z* = 4	0.60 × 0.56 × 0.44 mm

### Data collection

**Table:**

Enraf–Nonius CAD-4 diffractometer	*R*_int_ = 0.009
Radiation source: fine-focus sealed tube	θ_max_ = 25.2º
Monochromator: graphite	θ_min_ = 1.9º
*T* = 292(2) K	*h* = −2→13
ω/2θ scans	*k* = −6→14
Absorption correction: none	*l* = −3→25
3435 measured reflections	3 standard reflections
3116 independent reflections	every 200 reflections
1818 reflections with *I* > 2σ(*I*)	intensity decay: 0.9%

### Refinement

**Table:**

Refinement on *F*^2^	Hydrogen site location: inferred from neighbouring sites
Least-squares matrix: full	H-atom parameters constrained
*R*[*F*^2^ > 2σ(*F*^2^)] = 0.047	*w* = 1/[σ^2^(*F*_o_^2^) + (0.0695*P*)^2^] where *P* = (*F*_o_^2^ + 2*F*_c_^2^)/3
*wR*(*F*^2^) = 0.125	(Δ/σ)_max_ < 0.001
*S* = 0.97	Δρ_max_ = 0.19 e Å^−^^3^
3116 reflections	Δρ_min_ = −0.17 e Å^−^^3^
362 parameters	Extinction correction: SHELXL97 (Sheldrick, 2008), Fc^*^=kFc[1+0.001xFc^2^λ^3^/sin(2θ)]^-1/4^
Primary atom site location: structure-invariant direct methods	Extinction coefficient: 0.0090 (12)
Secondary atom site location: difference Fourier map	

### Special details

**Table:**

Geometry. All e.s.d.'s (except the e.s.d. in the dihedral angle between two l.s. planes) are estimated using the full covariance matrix. The cell e.s.d.'s are taken into account individually in the estimation of e.s.d.'s in distances, angles and torsion angles; correlations between e.s.d.'s in cell parameters are only used when they are defined by crystal symmetry. An approximate (isotropic) treatment of cell e.s.d.'s is used for estimating e.s.d.'s involving l.s. planes.
Refinement. Refinement of *F*^2^ against ALL reflections. The weighted *R*-factor *wR* and goodness of fit *S* are based on *F*^2^, conventional *R*-factors *R* are based on *F*, with *F* set to zero for negative *F*^2^. The threshold expression of *F*^2^ > 2σ(*F*^2^) is used only for calculating *R*-factors(gt) *etc*. and is not relevant to the choice of reflections for refinement. *R*-factors based on *F*^2^ are statistically about twice as large as those based on *F*, and *R*- factors based on ALL data will be even larger.

### Fractional atomic coordinates and isotropic or equivalent isotropic displacement parameters (Å^2^)

**Table:**

	*x*	*y*	*z*	*U*_iso_*/*U*_eq_	
O1	0.3112 (2)	−0.0655 (2)	0.43876 (11)	0.0676 (8)	
H1	0.2632	−0.1128	0.4442	0.101*	
O2	0.5639 (2)	0.28015 (19)	−0.04823 (12)	0.0593 (7)	
O3	0.3922 (2)	0.20169 (19)	−0.04048 (12)	0.0554 (7)	
O4	0.4470 (3)	0.4876 (2)	−0.14161 (14)	0.0785 (9)	
O5	0.4757 (3)	0.3145 (2)	−0.16352 (13)	0.0855 (10)	
C1	0.3267 (3)	0.0359 (3)	0.24624 (16)	0.0457 (9)	
C2	0.4278 (3)	0.0554 (3)	0.29194 (16)	0.0465 (9)	
H2	0.4800	−0.0049	0.2841	0.056*	
C3	0.4019 (3)	0.0475 (3)	0.36250 (16)	0.0495 (10)	
C4	0.3383 (3)	−0.0584 (3)	0.37360 (17)	0.0516 (10)	
H4	0.3922	−0.1166	0.3641	0.062*	
C5	0.2331 (4)	−0.0748 (3)	0.33319 (18)	0.0597 (11)	
H5A	0.1764	−0.0201	0.3425	0.072*	
H5B	0.1993	−0.1443	0.3421	0.072*	
C6	0.2661 (3)	−0.0687 (3)	0.26447 (16)	0.0527 (10)	
H6A	0.1970	−0.0764	0.2395	0.063*	
H6B	0.3164	−0.1285	0.2547	0.063*	
C7	0.3321 (4)	0.1428 (3)	0.38858 (18)	0.0654 (11)	
H7A	0.2538	0.1374	0.3749	0.098*	
H7B	0.3647	0.2089	0.3738	0.098*	
H7C	0.3346	0.1414	0.4333	0.098*	
C8	0.5167 (4)	0.0419 (3)	0.39817 (19)	0.0649 (12)	
H8A	0.5018	0.0303	0.4416	0.097*	
H8B	0.5578	0.1081	0.3929	0.097*	
H8C	0.5622	−0.0163	0.3822	0.097*	
C9	0.2352 (4)	0.1256 (3)	0.24777 (19)	0.0648 (12)	
H9A	0.1847	0.1145	0.2825	0.097*	
H9B	0.1915	0.1242	0.2099	0.097*	
H9C	0.2726	0.1939	0.2519	0.097*	
C10	0.4969 (4)	0.1536 (3)	0.27172 (17)	0.0579 (11)	
H10A	0.5552	0.1693	0.3027	0.069*	
H10B	0.4461	0.2152	0.2688	0.069*	
C11	0.5544 (3)	0.1341 (3)	0.20874 (16)	0.0537 (10)	
H11A	0.6105	0.0770	0.2132	0.064*	
H11B	0.5955	0.1985	0.1966	0.064*	
C12	0.4692 (3)	0.1036 (3)	0.15636 (17)	0.0478 (9)	
C13	0.3811 (3)	0.0197 (3)	0.18028 (16)	0.0472 (9)	
H13	0.4251	−0.0471	0.1837	0.057*	
C14	0.4064 (4)	0.2061 (3)	0.13466 (18)	0.0583 (11)	
H14A	0.3572	0.1892	0.1001	0.087*	
H14B	0.4620	0.2588	0.1221	0.087*	
H14C	0.3610	0.2341	0.1683	0.087*	
C15	0.5377 (3)	0.0506 (3)	0.10000 (17)	0.0472 (9)	
C16	0.4504 (3)	0.0129 (2)	0.05037 (16)	0.0450 (9)	
C17	0.3432 (3)	−0.0089 (3)	0.06576 (17)	0.0521 (10)	
H17	0.2946	−0.0305	0.0337	0.063*	
C18	0.2915 (3)	−0.0026 (3)	0.12919 (18)	0.0597 (11)	
H18A	0.2528	−0.0697	0.1383	0.072*	
H18B	0.2342	0.0540	0.1297	0.072*	
C19	0.6073 (4)	−0.0486 (3)	0.12069 (19)	0.0615 (11)	
H19A	0.5554	−0.1046	0.1332	0.092*	
H19B	0.6559	−0.0297	0.1551	0.092*	
H19C	0.6538	−0.0734	0.0867	0.092*	
C20	0.6231 (3)	0.1324 (3)	0.07259 (18)	0.0599 (11)	
H20A	0.5860	0.2021	0.0712	0.072*	
H20B	0.6886	0.1381	0.1004	0.072*	
C21	0.6667 (3)	0.1052 (3)	0.00753 (17)	0.0597 (11)	
H21A	0.7111	0.0394	0.0091	0.072*	
H21B	0.7168	0.1621	−0.0071	0.072*	
C22	0.5655 (3)	0.0913 (3)	−0.03835 (17)	0.0495 (10)	
C23	0.4897 (3)	−0.0026 (3)	−0.01690 (16)	0.0482 (9)	
H23	0.4199	0.0008	−0.0424	0.058*	
C24	0.5438 (4)	−0.1142 (3)	−0.02978 (18)	0.0640 (12)	
H24	0.6126	−0.1207	−0.0037	0.077*	
C25	0.5818 (4)	−0.1250 (3)	−0.0978 (2)	0.0719 (13)	
H25	0.5131	−0.1206	−0.1241	0.086*	
C26	0.6616 (4)	−0.0351 (4)	−0.1159 (2)	0.0772 (14)	
H26A	0.6821	−0.0427	−0.1595	0.093*	
H26B	0.7317	−0.0403	−0.0916	0.093*	
C27	0.6083 (4)	0.0742 (3)	−0.10584 (18)	0.0615 (11)	
H27A	0.5440	0.0829	−0.1342	0.074*	
H27B	0.6647	0.1290	−0.1157	0.074*	
C28	0.6415 (5)	−0.2339 (4)	−0.1107 (2)	0.114 (2)	
H28A	0.6680	−0.2357	−0.1530	0.171*	
H28B	0.5878	−0.2912	−0.1040	0.171*	
H28C	0.7058	−0.2422	−0.0830	0.171*	
C29	0.4606 (4)	−0.2040 (3)	−0.0109 (2)	0.0799 (14)	
H29A	0.3923	−0.1998	−0.0359	0.120*	
H29B	0.4406	−0.1962	0.0322	0.120*	
H29C	0.4969	−0.2724	−0.0173	0.120*	
C30	0.4959 (4)	0.1934 (3)	−0.04108 (16)	0.0479 (9)	
C31	0.5087 (4)	0.3793 (3)	−0.06002 (19)	0.0600 (11)	
H31A	0.5601	0.4377	−0.0491	0.072*	
H31B	0.4404	0.3851	−0.0343	0.072*	
C32	0.4757 (4)	0.3882 (4)	−0.1274 (2)	0.0605 (11)	
C33	0.4120 (5)	0.5073 (4)	−0.2062 (2)	0.0857 (15)	
H33A	0.4706	0.4813	−0.2346	0.103*	
H33B	0.3407	0.4698	−0.2151	0.103*	
C34	0.3961 (6)	0.6224 (4)	−0.2142 (2)	0.1113 (19)	
H34A	0.3488	0.6497	−0.1812	0.167*	
H34B	0.3593	0.6359	−0.2535	0.167*	
H34C	0.4696	0.6575	−0.2134	0.167*	

### Atomic displacement parameters (Å^2^)

**Table:**

	*U*^11^	*U*^22^	*U*^33^	*U*^12^	*U*^13^	*U*^23^
O1	0.076 (2)	0.083 (2)	0.0439 (16)	−0.0207 (18)	0.0043 (15)	0.0021 (13)
O2	0.0541 (16)	0.0551 (16)	0.0687 (18)	−0.0096 (14)	0.0000 (15)	0.0092 (13)
O3	0.0504 (17)	0.0527 (15)	0.0631 (18)	−0.0007 (14)	0.0040 (15)	0.0057 (13)
O4	0.100 (2)	0.0616 (18)	0.074 (2)	0.0112 (19)	−0.0051 (18)	0.0084 (15)
O5	0.124 (3)	0.073 (2)	0.0590 (18)	0.004 (2)	0.009 (2)	0.0028 (16)
C1	0.044 (2)	0.043 (2)	0.050 (2)	−0.0025 (19)	−0.0064 (19)	−0.0030 (16)
C2	0.049 (2)	0.0384 (19)	0.052 (2)	−0.0028 (19)	−0.002 (2)	−0.0017 (16)
C3	0.051 (2)	0.054 (2)	0.044 (2)	0.000 (2)	0.002 (2)	−0.0035 (17)
C4	0.053 (2)	0.053 (2)	0.049 (2)	−0.004 (2)	−0.001 (2)	0.0001 (18)
C5	0.060 (3)	0.061 (3)	0.057 (2)	−0.019 (2)	0.007 (2)	−0.004 (2)
C6	0.052 (2)	0.057 (2)	0.048 (2)	−0.015 (2)	−0.001 (2)	−0.0026 (18)
C7	0.073 (3)	0.062 (2)	0.062 (3)	−0.008 (2)	0.008 (2)	−0.013 (2)
C8	0.064 (3)	0.079 (3)	0.051 (2)	−0.012 (2)	−0.008 (2)	0.000 (2)
C9	0.066 (3)	0.063 (3)	0.065 (3)	0.010 (2)	−0.012 (2)	−0.004 (2)
C10	0.064 (3)	0.054 (2)	0.056 (2)	−0.014 (2)	−0.003 (2)	−0.0048 (19)
C11	0.049 (2)	0.058 (2)	0.054 (2)	−0.015 (2)	0.000 (2)	−0.0023 (18)
C12	0.048 (2)	0.046 (2)	0.049 (2)	−0.0072 (19)	−0.0035 (19)	0.0031 (17)
C13	0.041 (2)	0.046 (2)	0.055 (2)	−0.0082 (18)	−0.0107 (18)	−0.0003 (18)
C14	0.065 (3)	0.050 (2)	0.060 (2)	−0.005 (2)	0.002 (2)	0.0080 (19)
C15	0.043 (2)	0.045 (2)	0.053 (2)	−0.0041 (19)	−0.0038 (19)	0.0013 (18)
C16	0.050 (2)	0.0393 (19)	0.046 (2)	−0.0048 (19)	−0.0056 (19)	0.0028 (17)
C17	0.051 (3)	0.055 (2)	0.050 (2)	−0.013 (2)	−0.010 (2)	0.0068 (18)
C18	0.052 (2)	0.073 (3)	0.054 (3)	−0.016 (2)	−0.004 (2)	0.004 (2)
C19	0.059 (3)	0.067 (3)	0.058 (3)	0.006 (2)	−0.011 (2)	0.003 (2)
C20	0.049 (2)	0.078 (3)	0.053 (3)	−0.010 (2)	−0.009 (2)	−0.002 (2)
C21	0.046 (2)	0.069 (3)	0.064 (3)	−0.005 (2)	−0.001 (2)	0.003 (2)
C22	0.049 (2)	0.053 (2)	0.047 (2)	0.000 (2)	−0.004 (2)	0.0011 (17)
C23	0.045 (2)	0.052 (2)	0.048 (2)	0.0040 (19)	−0.0065 (19)	0.0016 (17)
C24	0.073 (3)	0.058 (2)	0.062 (3)	0.020 (2)	−0.017 (2)	−0.0056 (19)
C25	0.067 (3)	0.067 (3)	0.081 (3)	0.022 (3)	−0.015 (3)	−0.018 (2)
C26	0.064 (3)	0.100 (4)	0.068 (3)	0.014 (3)	0.008 (3)	−0.020 (3)
C27	0.051 (2)	0.076 (3)	0.057 (3)	0.001 (2)	0.006 (2)	−0.001 (2)
C28	0.129 (5)	0.093 (3)	0.121 (5)	0.045 (4)	0.004 (4)	−0.029 (3)
C29	0.103 (4)	0.050 (2)	0.087 (3)	0.002 (3)	−0.017 (3)	0.001 (2)
C30	0.049 (3)	0.059 (2)	0.035 (2)	−0.008 (2)	0.003 (2)	0.0008 (18)
C31	0.073 (3)	0.047 (2)	0.060 (3)	−0.008 (2)	0.003 (2)	0.0049 (19)
C32	0.062 (3)	0.055 (3)	0.064 (3)	−0.007 (2)	0.010 (2)	0.006 (2)
C33	0.090 (4)	0.105 (4)	0.063 (3)	0.018 (3)	−0.001 (3)	0.021 (3)
C34	0.142 (5)	0.093 (4)	0.100 (4)	0.030 (4)	0.009 (4)	0.036 (3)

### Geometric parameters (Å, °)

**Table:**

O1—C4	1.437 (4)	C15—C19	1.543 (5)
O1—H1	0.8200	C15—C16	1.545 (5)
O2—C30	1.348 (4)	C16—C17	1.317 (5)
O2—C31	1.415 (4)	C16—C23	1.528 (5)
O3—C30	1.210 (4)	C17—C18	1.491 (5)
O4—C32	1.319 (5)	C17—H17	0.9300
O4—C33	1.466 (5)	C18—H18A	0.9700
O5—C32	1.203 (5)	C18—H18B	0.9700
C1—C6	1.533 (5)	C19—H19A	0.9600
C1—C9	1.543 (5)	C19—H19B	0.9600
C1—C2	1.551 (5)	C19—H19C	0.9600
C1—C13	1.565 (5)	C20—C21	1.525 (5)
C2—C10	1.527 (5)	C20—H20A	0.9700
C2—C3	1.548 (5)	C20—H20B	0.9700
C2—H2	0.9800	C21—C22	1.544 (5)
C3—C4	1.531 (5)	C21—H21A	0.9700
C3—C8	1.540 (5)	C21—H21B	0.9700
C3—C7	1.544 (5)	C22—C30	1.510 (5)
C4—C5	1.513 (5)	C22—C23	1.535 (5)
C4—H4	0.9800	C22—C27	1.547 (5)
C5—C6	1.527 (5)	C23—C24	1.552 (5)
C5—H5A	0.9700	C23—H23	0.9800
C5—H5B	0.9700	C24—C25	1.531 (6)
C6—H6A	0.9700	C24—C29	1.534 (6)
C6—H6B	0.9700	C24—H24	0.9800
C7—H7A	0.9600	C25—C26	1.506 (6)
C7—H7B	0.9600	C25—C28	1.550 (5)
C7—H7C	0.9600	C25—H25	0.9800
C8—H8A	0.9600	C26—C27	1.512 (5)
C8—H8B	0.9600	C26—H26A	0.9700
C8—H8C	0.9600	C26—H26B	0.9700
C9—H9A	0.9600	C27—H27A	0.9700
C9—H9B	0.9600	C27—H27B	0.9700
C9—H9C	0.9600	C28—H28A	0.9600
C10—C11	1.528 (5)	C28—H28B	0.9600
C10—H10A	0.9700	C28—H28C	0.9600
C10—H10B	0.9700	C29—H29A	0.9600
C11—C12	1.546 (5)	C29—H29B	0.9600
C11—H11A	0.9700	C29—H29C	0.9600
C11—H11B	0.9700	C31—C32	1.501 (6)
C12—C14	1.544 (5)	C31—H31A	0.9700
C12—C13	1.551 (5)	C31—H31B	0.9700
C12—C15	1.592 (5)	C33—C34	1.457 (6)
C13—C18	1.538 (5)	C33—H33A	0.9700
C13—H13	0.9800	C33—H33B	0.9700
C14—H14A	0.9600	C34—H34A	0.9600
C14—H14B	0.9600	C34—H34B	0.9600
C14—H14C	0.9600	C34—H34C	0.9600
C15—C20	1.541 (5)		
			
C4—O1—H1	109.5	C18—C17—H17	116.6
C30—O2—C31	117.1 (3)	C17—C18—C13	112.9 (3)
C32—O4—C33	116.5 (4)	C17—C18—H18A	109.0
C6—C1—C9	107.1 (3)	C13—C18—H18A	109.0
C6—C1—C2	108.6 (3)	C17—C18—H18B	109.0
C9—C1—C2	113.3 (3)	C13—C18—H18B	109.0
C6—C1—C13	107.9 (3)	H18A—C18—H18B	107.8
C9—C1—C13	113.1 (3)	C15—C19—H19A	109.5
C2—C1—C13	106.7 (3)	C15—C19—H19B	109.5
C10—C2—C3	115.6 (3)	H19A—C19—H19B	109.5
C10—C2—C1	110.1 (3)	C15—C19—H19C	109.5
C3—C2—C1	117.5 (3)	H19A—C19—H19C	109.5
C10—C2—H2	103.9	H19B—C19—H19C	109.5
C3—C2—H2	103.9	C21—C20—C15	114.7 (3)
C1—C2—H2	103.9	C21—C20—H20A	108.6
C4—C3—C8	107.6 (3)	C15—C20—H20A	108.6
C4—C3—C7	110.7 (3)	C21—C20—H20B	108.6
C8—C3—C7	108.0 (3)	C15—C20—H20B	108.6
C4—C3—C2	107.5 (3)	H20A—C20—H20B	107.6
C8—C3—C2	108.7 (3)	C20—C21—C22	110.9 (3)
C7—C3—C2	114.1 (3)	C20—C21—H21A	109.5
O1—C4—C5	111.9 (3)	C22—C21—H21A	109.5
O1—C4—C3	108.1 (3)	C20—C21—H21B	109.5
C5—C4—C3	114.7 (3)	C22—C21—H21B	109.5
O1—C4—H4	107.3	H21A—C21—H21B	108.1
C5—C4—H4	107.3	C30—C22—C23	110.3 (3)
C3—C4—H4	107.3	C30—C22—C21	109.8 (3)
C4—C5—C6	110.2 (3)	C23—C22—C21	109.3 (3)
C4—C5—H5A	109.6	C30—C22—C27	104.6 (3)
C6—C5—H5A	109.6	C23—C22—C27	111.1 (3)
C4—C5—H5B	109.6	C21—C22—C27	111.6 (3)
C6—C5—H5B	109.6	C16—C23—C22	111.1 (3)
H5A—C5—H5B	108.1	C16—C23—C24	113.8 (3)
C5—C6—C1	113.9 (3)	C22—C23—C24	113.4 (3)
C5—C6—H6A	108.8	C16—C23—H23	105.9
C1—C6—H6A	108.8	C22—C23—H23	105.9
C5—C6—H6B	108.8	C24—C23—H23	105.9
C1—C6—H6B	108.8	C25—C24—C29	111.7 (3)
H6A—C6—H6B	107.7	C25—C24—C23	111.4 (3)
C3—C7—H7A	109.5	C29—C24—C23	110.6 (3)
C3—C7—H7B	109.5	C25—C24—H24	107.6
H7A—C7—H7B	109.5	C29—C24—H24	107.6
C3—C7—H7C	109.5	C23—C24—H24	107.6
H7A—C7—H7C	109.5	C26—C25—C24	111.1 (3)
H7B—C7—H7C	109.5	C26—C25—C28	109.3 (4)
C3—C8—H8A	109.5	C24—C25—C28	112.1 (4)
C3—C8—H8B	109.5	C26—C25—H25	108.1
H8A—C8—H8B	109.5	C24—C25—H25	108.1
C3—C8—H8C	109.5	C28—C25—H25	108.1
H8A—C8—H8C	109.5	C25—C26—C27	112.4 (3)
H8B—C8—H8C	109.5	C25—C26—H26A	109.1
C1—C9—H9A	109.5	C27—C26—H26A	109.1
C1—C9—H9B	109.5	C25—C26—H26B	109.1
H9A—C9—H9B	109.5	C27—C26—H26B	109.1
C1—C9—H9C	109.5	H26A—C26—H26B	107.9
H9A—C9—H9C	109.5	C26—C27—C22	113.0 (3)
H9B—C9—H9C	109.5	C26—C27—H27A	109.0
C2—C10—C11	110.7 (3)	C22—C27—H27A	109.0
C2—C10—H10A	109.5	C26—C27—H27B	109.0
C11—C10—H10A	109.5	C22—C27—H27B	109.0
C2—C10—H10B	109.5	H27A—C27—H27B	107.8
C11—C10—H10B	109.5	C25—C28—H28A	109.5
H10A—C10—H10B	108.1	C25—C28—H28B	109.5
C10—C11—C12	113.7 (3)	H28A—C28—H28B	109.5
C10—C11—H11A	108.8	C25—C28—H28C	109.5
C12—C11—H11A	108.8	H28A—C28—H28C	109.5
C10—C11—H11B	108.8	H28B—C28—H28C	109.5
C12—C11—H11B	108.8	C24—C29—H29A	109.5
H11A—C11—H11B	107.7	C24—C29—H29B	109.5
C14—C12—C11	108.6 (3)	H29A—C29—H29B	109.5
C14—C12—C13	110.3 (3)	C24—C29—H29C	109.5
C11—C12—C13	110.3 (3)	H29A—C29—H29C	109.5
C14—C12—C15	110.5 (3)	H29B—C29—H29C	109.5
C11—C12—C15	109.6 (3)	O3—C30—O2	121.2 (4)
C13—C12—C15	107.6 (3)	O3—C30—C22	127.2 (4)
C18—C13—C12	109.4 (3)	O2—C30—C22	111.5 (3)
C18—C13—C1	113.3 (3)	O2—C31—C32	110.7 (3)
C12—C13—C1	118.7 (3)	O2—C31—H31A	109.5
C18—C13—H13	104.7	C32—C31—H31A	109.5
C12—C13—H13	104.7	O2—C31—H31B	109.5
C1—C13—H13	104.7	C32—C31—H31B	109.5
C12—C14—H14A	109.5	H31A—C31—H31B	108.1
C12—C14—H14B	109.5	O5—C32—O4	124.6 (4)
H14A—C14—H14B	109.5	O5—C32—C31	124.5 (4)
C12—C14—H14C	109.5	O4—C32—C31	110.9 (4)
H14A—C14—H14C	109.5	C34—C33—O4	108.2 (4)
H14B—C14—H14C	109.5	C34—C33—H33A	110.1
C20—C15—C19	107.6 (3)	O4—C33—H33A	110.1
C20—C15—C16	111.2 (3)	C34—C33—H33B	110.1
C19—C15—C16	107.4 (3)	O4—C33—H33B	110.1
C20—C15—C12	109.7 (3)	H33A—C33—H33B	108.4
C19—C15—C12	112.1 (3)	C33—C34—H34A	109.5
C16—C15—C12	108.8 (3)	C33—C34—H34B	109.5
C17—C16—C23	119.7 (3)	H34A—C34—H34B	109.5
C17—C16—C15	120.7 (3)	C33—C34—H34C	109.5
C23—C16—C15	119.6 (3)	H34A—C34—H34C	109.5
C16—C17—C18	126.8 (4)	H34B—C34—H34C	109.5
C16—C17—H17	116.6		
			
C6—C1—C2—C10	174.9 (3)	C20—C15—C16—C23	36.8 (4)
C9—C1—C2—C10	−66.1 (4)	C19—C15—C16—C23	−80.7 (4)
C13—C1—C2—C10	58.9 (3)	C12—C15—C16—C23	157.7 (3)
C6—C1—C2—C3	−49.9 (4)	C23—C16—C17—C18	177.5 (3)
C9—C1—C2—C3	69.0 (4)	C15—C16—C17—C18	−0.7 (6)
C13—C1—C2—C3	−165.9 (3)	C16—C17—C18—C13	−7.3 (6)
C10—C2—C3—C4	−177.0 (3)	C12—C13—C18—C17	40.4 (4)
C1—C2—C3—C4	50.2 (4)	C1—C13—C18—C17	175.2 (3)
C10—C2—C3—C8	−60.8 (4)	C19—C15—C20—C21	75.6 (4)
C1—C2—C3—C8	166.4 (3)	C16—C15—C20—C21	−41.8 (4)
C10—C2—C3—C7	59.8 (4)	C12—C15—C20—C21	−162.2 (3)
C1—C2—C3—C7	−73.0 (4)	C15—C20—C21—C22	56.4 (4)
C8—C3—C4—O1	64.5 (4)	C20—C21—C22—C30	59.1 (4)
C7—C3—C4—O1	−53.3 (4)	C20—C21—C22—C23	−62.0 (4)
C2—C3—C4—O1	−178.6 (3)	C20—C21—C22—C27	174.6 (3)
C8—C3—C4—C5	−170.0 (3)	C17—C16—C23—C22	137.4 (4)
C7—C3—C4—C5	72.2 (4)	C15—C16—C23—C22	−44.5 (4)
C2—C3—C4—C5	−53.0 (4)	C17—C16—C23—C24	−93.2 (4)
O1—C4—C5—C6	−179.0 (3)	C15—C16—C23—C24	85.0 (4)
C3—C4—C5—C6	57.5 (4)	C30—C22—C23—C16	−66.1 (4)
C4—C5—C6—C1	−56.1 (4)	C21—C22—C23—C16	54.8 (4)
C9—C1—C6—C5	−71.5 (4)	C27—C22—C23—C16	178.4 (3)
C2—C1—C6—C5	51.2 (4)	C30—C22—C23—C24	164.3 (3)
C13—C1—C6—C5	166.4 (3)	C21—C22—C23—C24	−74.9 (4)
C3—C2—C10—C11	159.2 (3)	C27—C22—C23—C24	48.8 (4)
C1—C2—C10—C11	−64.7 (4)	C16—C23—C24—C25	−179.9 (4)
C2—C10—C11—C12	56.7 (4)	C22—C23—C24—C25	−51.7 (4)
C10—C11—C12—C14	76.7 (4)	C16—C23—C24—C29	55.2 (4)
C10—C11—C12—C13	−44.3 (4)	C22—C23—C24—C29	−176.5 (3)
C10—C11—C12—C15	−162.5 (3)	C29—C24—C25—C26	178.9 (3)
C14—C12—C13—C18	55.5 (4)	C23—C24—C25—C26	54.6 (5)
C11—C12—C13—C18	175.4 (3)	C29—C24—C25—C28	−58.6 (5)
C15—C12—C13—C18	−65.1 (4)	C23—C24—C25—C28	177.2 (4)
C14—C12—C13—C1	−76.6 (4)	C24—C25—C26—C27	−56.9 (5)
C11—C12—C13—C1	43.4 (4)	C28—C25—C26—C27	178.9 (4)
C15—C12—C13—C1	162.9 (3)	C25—C26—C27—C22	55.1 (5)
C6—C1—C13—C18	62.5 (4)	C30—C22—C27—C26	−169.3 (3)
C9—C1—C13—C18	−55.8 (4)	C23—C22—C27—C26	−50.2 (5)
C2—C1—C13—C18	179.1 (3)	C21—C22—C27—C26	72.1 (4)
C6—C1—C13—C12	−167.1 (3)	C31—O2—C30—O3	−5.0 (5)
C9—C1—C13—C12	74.6 (4)	C31—O2—C30—C22	171.6 (3)
C2—C1—C13—C12	−50.6 (4)	C23—C22—C30—O3	−15.3 (5)
C14—C12—C15—C20	57.2 (4)	C21—C22—C30—O3	−135.9 (4)
C11—C12—C15—C20	−62.4 (4)	C27—C22—C30—O3	104.2 (4)
C13—C12—C15—C20	177.6 (3)	C23—C22—C30—O2	168.3 (3)
C14—C12—C15—C19	176.7 (3)	C21—C22—C30—O2	47.8 (4)
C11—C12—C15—C19	57.1 (4)	C27—C22—C30—O2	−72.1 (4)
C13—C12—C15—C19	−62.8 (4)	C30—O2—C31—C32	−80.2 (4)
C14—C12—C15—C16	−64.7 (3)	C33—O4—C32—O5	0.6 (7)
C11—C12—C15—C16	175.7 (3)	C33—O4—C32—C31	−179.6 (4)
C13—C12—C15—C16	55.8 (3)	O2—C31—C32—O5	13.2 (6)
C20—C15—C16—C17	−145.1 (4)	O2—C31—C32—O4	−166.6 (3)
C19—C15—C16—C17	97.4 (4)	C32—O4—C33—C34	−173.9 (4)
C12—C15—C16—C17	−24.1 (4)		

### Hydrogen-bond geometry (Å, °)

**Table:**

*D*—H···*A*	*D*—H	H···*A*	*D*···*A*	*D*—H···*A*
O1—H1···O3^i^	0.82	2.14	2.944 (4)	165

Symmetry codes: (i) −*x*+1/2, −*y*, *z*+1/2.
